# Supplementary material for: Valence band electronic structure of the van der Waals ferromagnetic insulators: VI3 and CrI3
Source: Sci Rep. 2020 Sep 24;10:15602. doi: 10.1038/s41598-020-72487-5 (PMC7515918; doi:10.1038/s41598-020-72487-5)
Supplement: Supplementary file 1 — Supplementary file1 [file 41598_2020_72487_MOESM1_ESM.pdf]

Valence band electronic structure of the van der Waals ferromagnetic insulators: VI_3 and CrI_3

Asish K. Kundu[1,*], Yu Liu[1,†], C. Petrovic[1], and T. Valla[1,‡]

[1]Condensed Matter Physics and Materials Science Department, Brookhaven National Laboratory, Upton, New York 11973, USA

[†] present address: Los Alamos National Laboratory, MS K764, Los Alamos NM 87545
* akundu@bnl.gov
[‡] valla@bnl.gov

Comparison of experimental and theoretical band structure in VI_3:

Fig. S1 compares the experimental band structure obtained in ARPES with the theoretical band dispersions of VI_3 [23]. The red- and blue lines represent the spin-up and spin-down bands. Although the general features look very similar, the individual spin-up and spin-down bands are not resolved in the experiments. This might be due to the relatively large state widths in the experiment, but it might also indicate that the calculations are problematic as they do not account for the spin-orbit coupling, the essential parameter to properly describe this system.

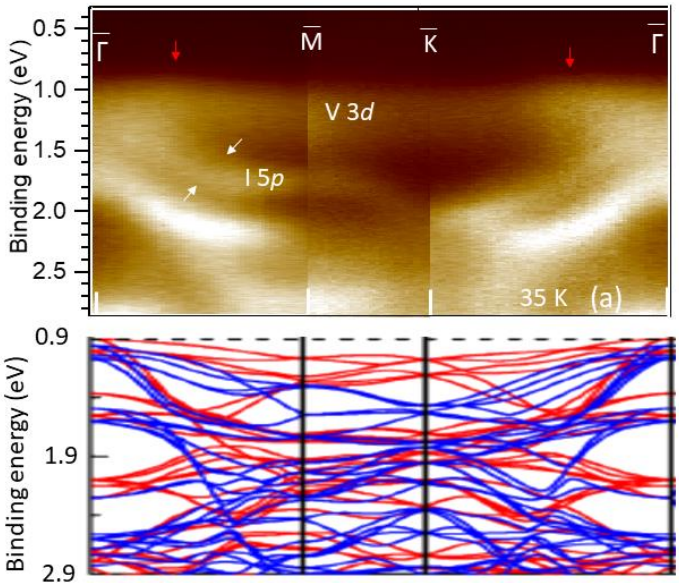

Fig. S1. Comparison of experimental and theoretical band dispersions in VI_3. Top panel is the experimental electronic structure, measured at 35 K and the bottom panel represents the calculated bands, reproduced from Tian *et al.* [23]. The spin-up and spin-down bands are plotted in red and blue, respectively.

Position of VBM in VI_3:

In, Fig. S2., a set of EDCs of photoemission intensity, measured along the line shown in the inset is shown. The dispersion of the V 3*d* band is represented by the blue dotted curve. It is clear that the position of VBM is not exactly at $\bar{\Gamma}$ but at around k_{II} = 0.23Å^{-1}.

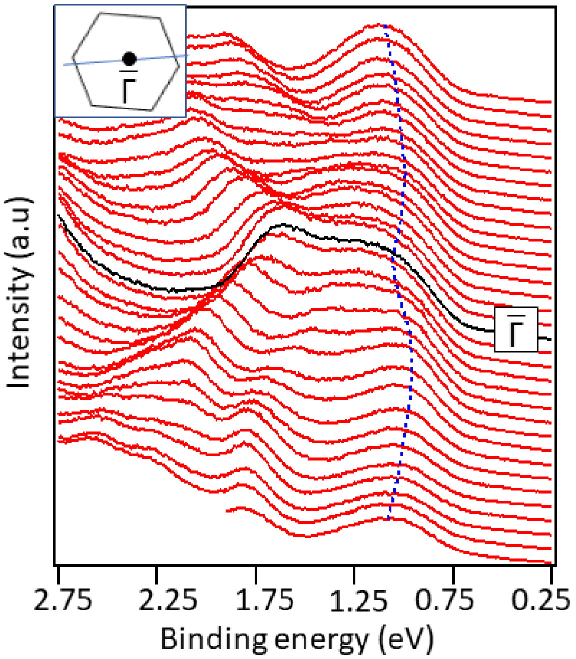

Fig. S2. EDCs of photoemission intensity extracted from Fig. 4(a), measured along the line shown in the inset. EDCs are offset in y-direction for clarity. The dispersion of the V 3d band is shown by the dotted curve (blue).

Electronic structure below and above magnetic transition in VI_3:

Figure S3 shows Lorentzian fits of the MDCs at 1.7 eV binding energy from spectra taken at 300 and 35 K as shown in Fig. 4(c) in the manuscript. The separation between peaks 4 and 5 increases from 0.134 ± 0.004 Å^{-1} to 0.160 ± 0.004 Å^{-1} with lowering the temperature from 300 K to 35 K. This might be an indication of an additional contribution of the exchange splitting to the separation of these two bands in the FM state.

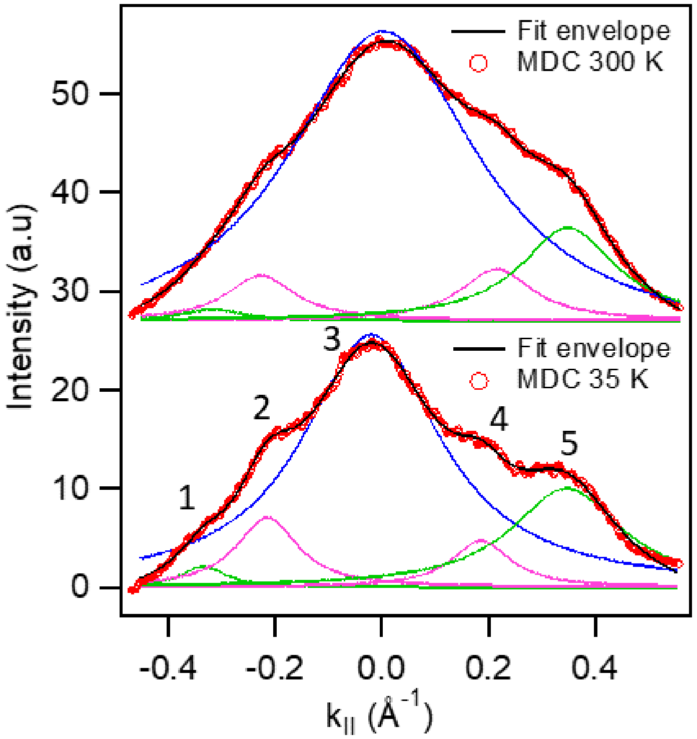

Fig. S3. Momentum distribution curves (MDCs) at 1.7 eV binding energy for two different temperatures, 300 K and 35 K. Five Lorentzian peaks and a constant background were used to fit the data. A slight increase in separation (Δk=0.026±0.004) between peaks 4 and 5 was detected on cooling from 300 to 35 K.
